# Academic training of authors publishing in high-impact epidemiology and clinical journals

**DOI:** 10.1371/journal.pone.0271159

**Published:** 2022-07-29

**Authors:** Amanda Sullivan, Eleanor J. Murray, Laura Corlin

**Affiliations:** 1 Department of Public Health and Community Medicine, Tufts University School of Medicine, Boston, Massachusetts, United States of America; 2 Department of Epidemiology, Boston University School of Public Health, Boston, Massachusetts, United States of America; 3 Department of Civil and Environmental Engineering, Tufts University School of Engineering, Medford, Massachusetts, United States of America; Universita degli Studi di Brescia, ITALY

## Abstract

**Background:**

To inform training program development and curricular initiatives, quantitative descriptions of the disciplinary training of research teams publishing in top-tier clinical and epidemiological journals are needed. Our objective was to assess whether interdisciplinary academic training and teamwork of authors publishing original research in 15 top-tier journals varied by year of publication (2000/2010/2020), type of journal (epidemiological/general clinical/specialty clinical), corresponding author gender, and time since the corresponding author completed formal training relative to the article publication date (<5/≥5 years).

**Methods and findings:**

We invited corresponding authors of original research articles to participate in an online survey (n = 103; response rate = 8.3% of 1240 invited authors). In bivariate analyses, year of publication, type of journal, gender, and recency of training were not significantly associated with interdisciplinary team composition, whether a co-author with epidemiological or biostatistical training was involved in any research stage (design/analysis/interpretation/reporting), or with participants’ confidence in their own or their co-authors epidemiological or biostatistical expertise (p > 0.05 for each comparison). Exceptions were participants with more recent epidemiological training all had co-author(s) with epidemiological training contribute to study design and interpretation, and participants who published in 2020 were more likely to report being extremely confident in their epidemiological abilities.

**Conclusions:**

This study was the first to quantify interdisciplinary training among research teams publishing in epidemiological and clinical journals. Our quantitative results show research published in top-tier journals generally represents interdisciplinary teamwork and that interdisciplinary training may provide publication type options. Our qualitative results show researchers view interdisciplinary training favorably.

## Introduction

We rely on high-quality science to help address the myriad clinical and public health challenges facing society. Two of the primary ways in which we uphold the rigor of scientific literature are through formal academic training and interdisciplinary teamwork. Although the necessary academic training of research teams is specific to each research question, clinician-scholars publishing original research in the clinical and epidemiological literature often require at least a basic understanding of epidemiology and biostatistics to effectively collaborate in interdisciplinary teams. For this reason, and because of the clinical value in understanding the basic methods underlying clinical literature [1–3], introductory epidemiology and biostatistics concepts comprise 4–6% of the Step 1 examination questions for the United States Medical Licensing Exam [4]. Additionally, medical schools and funding organizations support innovative interdisciplinary training programs for clinician-scholars to learn research methods [5–13]. As with clinician-scholar training programs, accredited public health training typically includes at least introductory coursework in epidemiology and biostatistics.

Complementing these trends in training, the academic literature has also seen an increase in team science and increased attention to authorship practices. The mean number of authors for articles in medical journals increased by 23% between 1995 and 2005 [14], and similar trends have been observed in terms of the number of authors on research articles in the health science literature more broadly [15, 16]. Although trends for gender of authors (including the gender of authors in specific authorship positions) has been investigated in the biomedical and epidemiological literature [17–19], little is known about the training experience of research teams publishing in top-tier clinical and epidemiological journals–either now or historically. Furthermore, to our knowledge, no study has examined how factors such as journal type, publication year, gender of the corresponding author, or recency of the corresponding author’s training relate to the disciplinary training of research teams.

To address these gaps in the literature, we administered a survey to quantify how formal interdisciplinary training and teamwork varied by year of publication, type of journal, gender, and time since training. Our primary hypotheses were that interdisciplinary training and interdisciplinary teamwork would be more common among (1) more recently published articles than among older articles, (2) articles published in epidemiological journals than clinical journals, (3) articles with women corresponding authors than men corresponding authors, and (4) articles with corresponding authors who completed training within five years of the date of publication compared to less recent training. We also hypothesized that gender and year of publication would be associated with authors’ confidence in their own and their co-authors’ understanding of relevant epidemiological and biostatistical concepts.

## Methods

### Study population and participant recruitment

**Fig 1:**
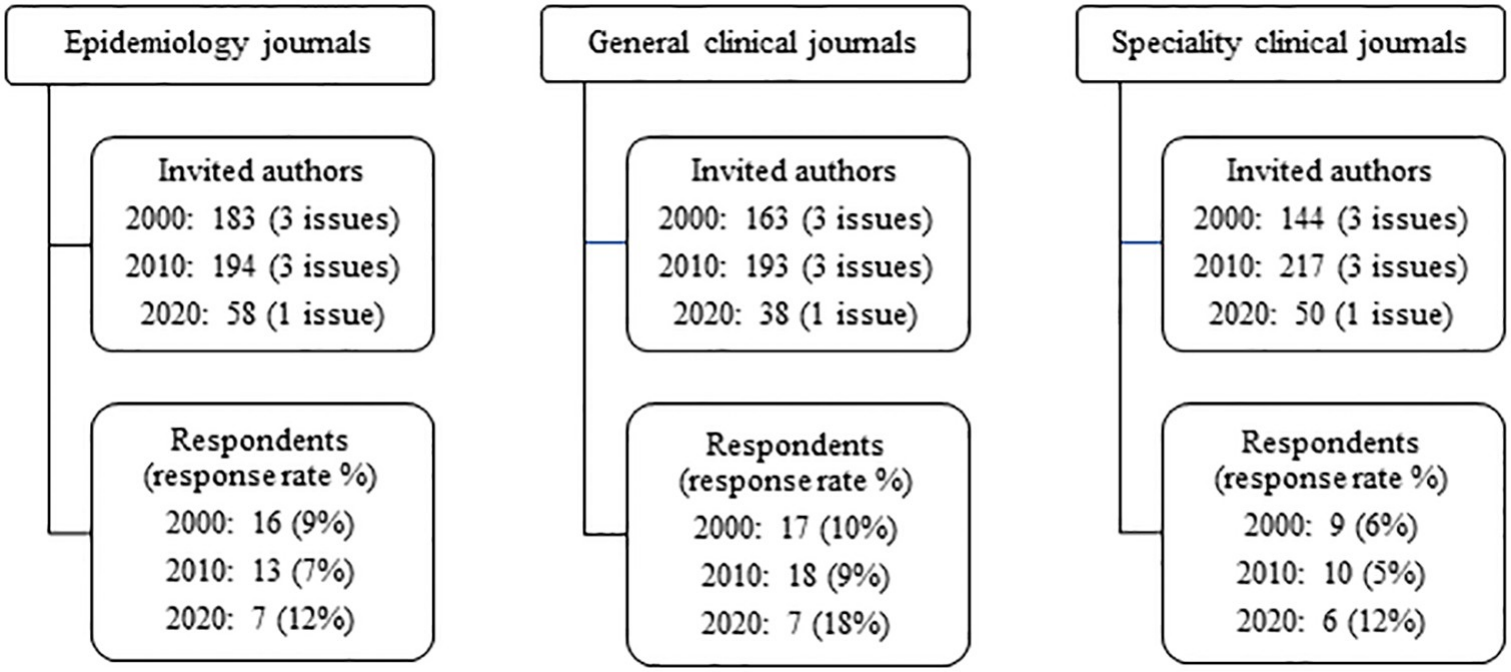
Participant selection.

Our target population included scholars publishing original research in leading English-language clinical and epidemiological journals. We selected 15 journals based on high impact factor (median impact factor for selected journals = 13.9 based on Web of Science Group; higher impact factors correspond to higher citation counts per article though ‘good’ impact factors are field-specific [20]) and positive reputation (e.g., journals associated with leading professional societies). Of these 15 journals, five were general clinical journals (chosen to represent clinical research in people of all ages; all in the top 5% of medical journals based on Scimago Journal Rankings [21]), five were specialty clinical journals (chosen to represent clinical research in a variety of specialties for people of all ages; all in the top three journals listed in their given specialty based on Scimago Journal Rankings [21]), and five were epidemiology journals (chosen to represent epidemiology as a whole, rather than a specific sub-discipline; all in the top third of epidemiology journals based on Scimago Journal Rankings [21]). Journal names will remain unspecified for confidentiality reasons and for compliance with the Institutional Review Board protocol. We selected three issues (same issue number distribution for each journal) from each of these 15 journals for each of the 2000 and 2010 volumes. We selected only one issue from the 2020 volume due to severe COVID-19-related delays for 2020 issues for many journals. One target 2000 journal issue did not have original research articles and was not included. In total, there were 1240 original research articles targeted (Fig 1). By contacting the corresponding authors of 1240 original research articles, we estimate that that we contacted approximately 1% of corresponding authors for all original research articles published in top clinical and epidemiological journals in those three years (assuming journals have an average of nine issues per year; that our sample of journals represents 33% of top epidemiology journals, 5% of top medical journals, and 2% of top medical specialty journals for the given specialties; and that 5% of articles in this set have overlapping corresponding authors).

Between January 8, 2020 and April 30, 2020, we invited each of the 1240 corresponding authors from the target articles to complete an online survey. The email invitation and reminder text are given in the (S1 and S2 Files in S1 Text). We used the corresponding author’s email address listed in the publication whenever possible because of the frequent practice of listing the author most familiar with the work as corresponding author. If we received an automatic response that the email address was no longer valid (n = 377) or if an email address was not listed on the publication (n = 157, all for papers published in 2000), we used public websites such as departmental lists of faculty and LinkedIn to identify a current email address. If we did not find a valid email address for the corresponding author or if the corresponding author was deceased (n = 30), we used public websites to find an email address for the first or last author (in that order, depending on who was listed as the corresponding author). For simplicity, we refer to all participants as the corresponding author of their respective articles. Overall, the response rate was 8.3% (Fig 1). All participants provided written informed consent through the online survey. The Tufts Social, Behavioral, & Educational Research Institutional Review Board approved the study (protocol 1910004).

### Survey instrument

We developed an online Qualtrics survey and pilot tested it within the larger research team of one author (LC); no member of the research team involved in pilot testing had helped develop the survey or was otherwise involved in the project in any way. We have previously developed and used surveys to assess epidemiology methods training [22]. The full survey instrument we used for the present analysis is given in the (S3 File in S1 Text).

#### Data about participants

Participants reported their gender, year of birth, level of education as of the time their paper was published, year their most recent degree had been awarded as of the time their paper was published, and country of training. We also asked participants: “As of the time your paper was published, had you ever received formal training in epidemiology [biostatistics; assessed separately]? (select all that apply: at least a full semester of an undergraduate-level course, at least a full semester of a graduate-level course, a workshop, an online class, training in another place, no unsure, prefer not to answer).” Participants that stated they had at least a full semester course (undergraduate or graduate) were considered to have formal training. Participants with formal training were asked in what year they had most recently obtained formal training in that area. Participants who selected ‘no,’ ‘unsure,’ or ‘prefer not to answer’ to the formal training question were asked: “As of the time your paper was published, would you have benefited from formal training (at least a full semester course) in epidemiology [biostatistics; separate question]?” Regardless of whether participants reported formal training experience, we asked them to rate their confidence in their personal ability to appropriately apply epidemiological [biostatistical; separate question] concepts relevant to their paper (not at all, not very, somewhat, very, or extremely confident). Finally, we asked an open-ended question: “As a reminder, we are trying to learn about the training of authors publishing epidemiologic research. Do you have any final thoughts you would like to share with us on this topic?”

#### Data about participants’ co-authors

In addition to asking about participants’ own training, we asked them to report on their co-authors’ training, role in the project, and abilities. We asked participants to exclude their own training when answering this set of questions, and to define formal training in this context as having at least two full semester courses in the subject (to select for co-authors with more advanced training/specific expertise). We first asked: “As of the time your paper was published, were any of your co-authors formally trained in epidemiology [biostatistics; separate question]?” If participants indicated that they had a co-author formally trained, we asked them to select all of the stages of the project that the co-author participated (design, analysis, interpretation, reporting, other). We asked similar questions about clinical training, but we only included co-authors with a clinical degree. For participants with clinician co-authors, we asked “did any of your clinician co-authors have formal training in epidemiology and/or biostatistics? (yes, no, unsure).” For all participants, we asked: “Prior to submitting your paper for publication in the journal it was accepted in, did anyone formally trained in epidemiology and/or biostatistics review your paper? (yes, no, unsure).” Finally, we asked participants to rate their confidence in their co-authors’ ability to apply epidemiological and biostatistical concepts [separate question] relevant to their paper (not at all, not very, somewhat, very, or extremely confident).

### Statistical analysis

We examined summary statistics for each variable, and for each variable stratified by type of publication, year of publication, gender of the participant, and years between publication of the article and time when the participant last received formal training (<5/≥5 years; separate variables for having at least a full semester course of epidemiology and biostatistics). Our analyses focused primarily on whether individuals had any epidemiology or biostatistics training, had interdisciplinary training (defined as training in clinical practice and formal training in either epidemiology or biostatistics), or worked with interdisciplinary teams (defined as teams with at least one author who had formal training in each of clinical practice, epidemiology, and biostatistics). If the participant reported that they last received training in a year prior to their birth (n = 9), we excluded them from all analyses focused on recency of training. We assessed bivariate associations among pairs of variables using linear regression (for associations with continuous normally distributed variables like age at publication), logistic regression (for associations with dichotomized variables like gender), and chi-square tests (for associations with categorical variables like journal type). For all analyses, we considered associations significant if p < 0.05. Data were analyzed using Stata MP 16.1 for Windows (Stata Corp, College Station, Texas). For the open-ended question, we reported emergent themes.

## Results

### Participant characteristics

**Fig 2:**
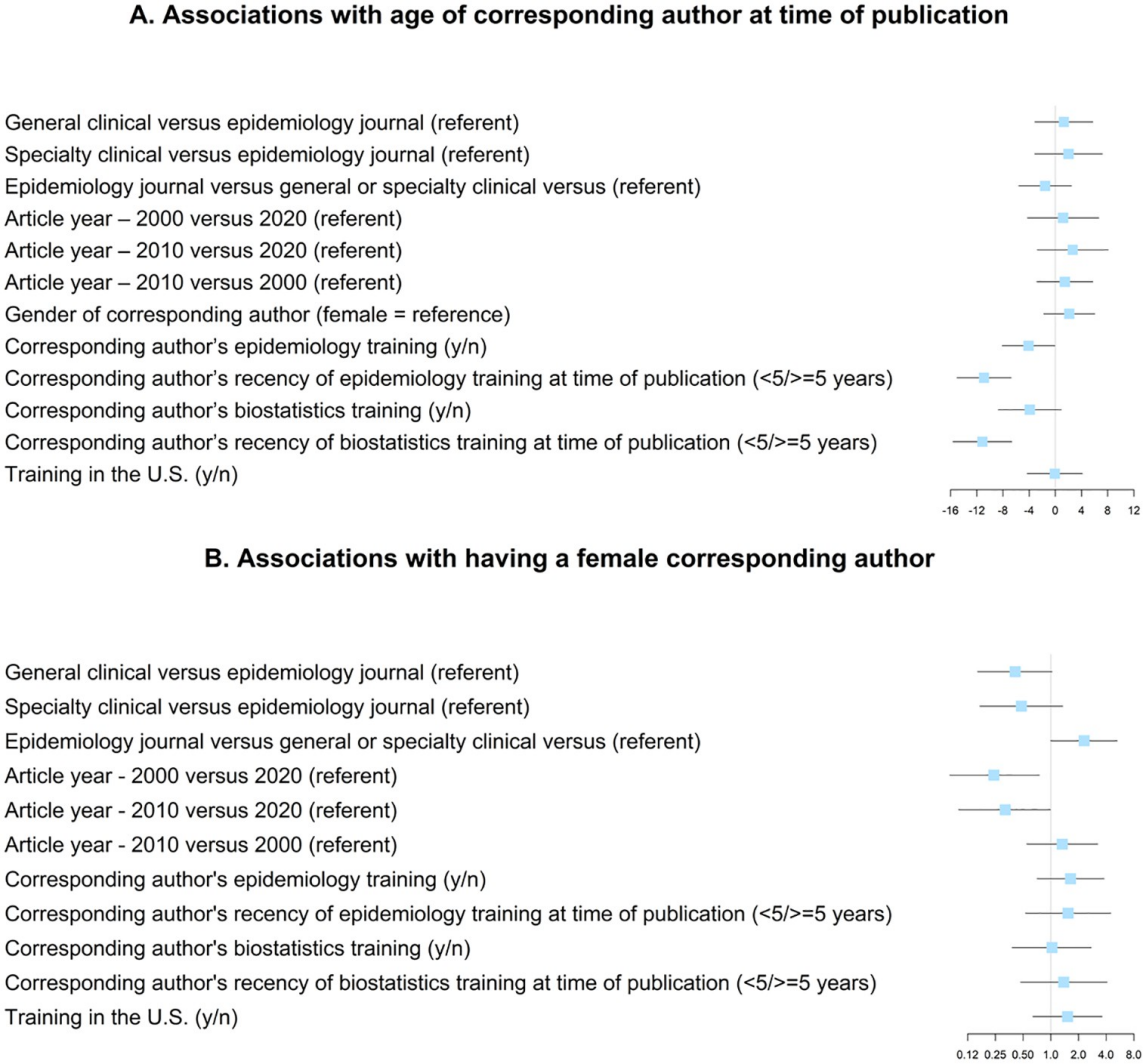
Bivariate associations with (A) age of corresponding author at time of publication and (B) having a female corresponding author. Boxes represent betas (panel A) or odds ratios (panel B). Lines represent 95% confidence intervals.

Overall, the mean age of our 103 participants at the time of the survey was 57.9 years (standard deviation = 12.2), 55% of participants were men, and 68% had received training in the U.S., 43% had a clinical degree, and 37% had a Doctor of Medicine (Table 1). With four exceptions, there were no statistically significant bivariate associations (p < 0.05) among journal type, publication year, gender, and age at the time of publication, or with any of training in the U.S., training in epidemiology, training in biostatistics, recency of epidemiology training, or recency of biostatistics training (Fig 2; S1 Table in S1 Text). The exceptions were that: (1) participants were more likely to be women if their article was published in 2020 than in 2000 (odds ratio (OR) = 4.2, 95% confidence interval (CI) = 1.3–13.2) or 2010 (OR = 3.2, 95% CI = 1.0–9.9); (2) participants were more likely to be women if their article was published in an epidemiological journal than in a general or specialty medical journal (OR = 2.3, 95% CI = 1.0–5.3); (3) individuals with formal epidemiological training were four years younger, on average, than individuals without formal epidemiological training (95% CI = 0.1–8 years younger); and (4) the mean age of individuals that had last received training in epidemiology or biostatistics within five years of publication was 11 years younger than individuals who received training at least five years before publication (95% CI for epidemiology = 7–15 years younger; 95% CI for biostatistics = 7–16 years younger). Notably, we also observed that the proportion of women corresponding authors increased over time (36% in 2000, 43% in 2010, and 70% in 2020; χ^2^ p = 0.037).

**Table 1:** Participant characteristics.

	Overall	Gender		Publication year			Journal type			Time since epidemiology training		Time since biostatistics training	
		female	male	2000	2010	2020	epidemiology	general clinical	specialty clinical	<5 years	≥5 years	<5 years	≥5 years
	(n = 103)	(n = 46)	(n = 56)	(n = 42)	(n = 41)	(n = 20)	(n = 36)	(n = 42)	(n = 25)	(n = 20)	(n = 44)	(n = 17)	(n = 59)
Gender, n (%)													
Female	46 (45)			15 (36)	17 (43)	14 (70)	21 (58)	15 (37)	10 (40)	11 (55)	19 (44)	9 (53)	26 (45)
Male	56 (55)			27 (64)	23 (58)	6 (30)	15 (42)	26 (63)	15 (60)	9 (45)	24 (56)	8 (47)	32 (55)
Age at time of survey (years), mean (standard deviation (SD))	57.9 (12.2)	55.1 (12.3)	60.1 (11.9)	65.3 (8.0)	56.7 (10.9)	44.1 (10.1)	56.9 (13.9)	58.6 (11.7)	58.0 (10.7)	48.0 (11.0)	59.7 (11.0)	48.3 (10.1)	59.5 (11.5)
Age at time of publication (years), mean (SD)	45.6 (9.6)	44.4 (9.7)	46.6 (9.6)	45.3 (8.0)	46.7 (10.9)	44.1 (10.1)	44.6 (9.3)	45.9 (10.5)	46.7 (8.7)	37.0 (5.6)	47.9 (8.4)	35.9 (4.9)	47.1 (8.9)
Most advanced degree, n (%)													
Master’s	11 (11)	6 (13)	5 (9)	4 (10)	5 (12)	2 (10)	5 (14)	2 (5)	4 (16)	4 (20)	2 (5)	5 (29)	4 (7)
PhD	42 (41)	21 (46)	21 (38)	15 (36)	15 (37)	12 (60)	19 (53)	14 (33)	9 (36)	7 (35)	15 (34)	4 (24)	25 (42)
MD	24 (23)	9 (20)	14 (25)	10 (24)	10 (24)	4 (20)	1 (3)	14 (33)	9 (36)	6 (30)	13 (30)	6 (35)	16 (27)
PhD/MD	14 (14)	5 (11)	9 (16)	9 (21)	3 (7)	2 (10)	5 (14)	8 (19)	1 (4)	1 (5)	6 (14)	1 (6)	4 (7)
Epidemiology training, n (%)	68 (66)	33 (72)	34 (61)	25 (60)	26 (63)	17 (85)	25 (69)	26 (62)	17 (68)			13 (76)	50 (85)
Biostatistics training, n (%)	82 (80)	37 (80)	44 (80)	34 (81)	32 (80)	16 (80)	28 (78)	33 (79)	21 (88)	20 (100)	42 (95)		
Country or continent of training (ever), n (%)													
United States	68 (68)	32 (73)	35 (64)	25 (61)	31 (78)	12 (63)	23 (66)	26 (63)	19 (79)	15 (75)	33 (77)	13 (81)	43 (74)
Europe	34 (34)	11 (25)	23 (42)	18 (44)	10 (25)	6 (32)	14 (40)	15 (37)	5 (21)	6 (30)	11 (26)	4 (25)	16 (28)
Other	8 (8)	5 (11)	3 (5)	1 (2)	4 (10)	3 (16)	4 (11)	2 (5)	2 (8)	0 (0)	6 (14)	0 (0)	6 (10)
Confidence in personal ability to apply epidemiology concepts to paper, n (%)													
Not at all/not very	10 (10)	3 (7)	7 (13)	5 (12)	5 (13)	0 (0)	1 (3)	7 (18)	2 (8)	0 (0)	0 (0)	3 (18)	4 (7)
Somewhat	7 (7)	6 (13)	1 (2)	2 (5)	1 (3)	4 (20)	1 (3)	4 (10)	2 (8)	2 (10)	0 (0)	1 (6)	2 (3)
Very	40 (40)	18 (40)	22 (42)	20 (49)	17 (45)	3 (15)	18 (50)	13 (33)	9 (38)	7 (35)	18 (42)	6 (35)	24 (41)
Extremely	42 (42)	18 (40)	23 (43)	14 (34)	15 (39)	13 (65)	16 (44)	15 (38)	11 (46)	11 (55)	25 (58)	7 (41)	29 (49)
Confidence in personal ability to apply biostatistics concepts to paper, n (%)													
Not at all/not very	4 (4)	1 (2)	3 (6)	1 (2)	2 (5)	1 (5)	2 (6)	2 (5)	0 (0)	1 (5)	0 (0)	1 (6)	2 (3)
Somewhat	21 (21)	10 (22)	11 (21)	10 (24)	5 (13)	6 (30)	4 (11)	9 (23)	8 (33)	3 (15)	11 (26)	4 (24)	13 (22)
Very	42 (42)	19 (42)	23 (43)	17 (41)	18 (47)	7 (35)	16 (44)	16 (41)	10 (42)	10 (50)	15 (35)	9 (53)	21 (36)
Extremely	32 (32)	15 (33)	16 (30)	13 (32)	13 (34)	6 (30)	14 (39)	12 (31)	6 (25)	6 (30)	17 (40)	3 (18)	23 (39)

### Interdisciplinary training

Most participants had interdisciplinary training (S2 Table in S1 Text). For example, 73% of the 44 participants with clinical training also had formal epidemiological or biostatistical training (n = 32) and 64% of clinicians had formal training in both epidemiology and biostatistics (n = 28). However, among the 18 participants in the full sample without either epidemiological or biostatistical training, only five indicated that they would have benefitted from epidemiological training and only two indicated that they would have benefited from biostatistical training.

Although none of publication year, gender, or recency of training was significantly associated with interdisciplinary training (p > 0.05 for each bivariate comparison; Table 2), interdisciplinary training seemed to affect the types of journals in which participants published. This was especially true for the clinical journals: participants who published in general clinical journals and specialty clinical journals were more likely than participants who published in epidemiological journals to have interdisciplinary training (OR = 4.9, 95% CI = 1.5–16.5 for general clinical journals; OR = 3.8, 95% CI = 0.99–14.3 for specialty clinical journals; referent group = epidemiological journals). These general trends held when controlling for publication year and gender (95% CI for general clinical versus epidemiological = 1.2–14.6; 95% CI for specialty clinical versus epidemiological = 0.92–13.9). Additionally, interdisciplinary training seemed to provide options to clinicians for journal outlets; five of the 32 (16%) clinicians with formal training in epidemiology or biostatistics published their articles in epidemiological journals compared to only one of the 11 clinicians (9%) without this training. Furthermore, journals tended to have corresponding authors with formal training in fields corresponding to the journal type. Of the 67 articles published in a clinical journal, 38 (57%) had a corresponding author with clinical training. Similarly, of the 36 articles published in an epidemiological journal, 29 (81%) had a corresponding author with formal epidemiological or biostatistical training.

**Table 2:** Bivariate associations between independent variables and interdisciplinary training^a^ and teamwork^b^.

Independent variable	Interdisciplinary training	Interdisciplinary teamwork
	odds ratio (95% confidence interval)	odds ratio (95% confidence interval)
General clinical versus epidemiology journal (referent)	4.92 (1.47, 16.54)*	1.79 (0.69, 4.69)
Specialty clinical versus epidemiology journal (referent)	3.76 (0.99, 14.33)	2.02 (0.65, 6.28)
Epidemiology journal versus general or specialty clinical versus (referent)	0.22 (0.07, 0.71)*	0.53 (0.22, 1.27)
Article year – 2000 versus 2020 (referent)	1.42 (0.39, 5.18)	0.83 (0.25, 2.80)
Article year – 2010 versus 2020 (referent)	1.86 (0.52, 6.67)	0.64 (0.19, 2.14)
Article year – 2010 versus 2000 (referent)	1.31 (0.51, 3.39)	0.77 (0.30, 1.96)
Gender of corresponding author (female/male)	0.51 (0.20, 1.29)	1.00 (0.43, 2.33)
Age of corresponding author at time of publication	1.02 (0.98, 1.07)	0.97 (0.93, 1.02)
Corresponding author’s epidemiology training (y/n)	NA	4.48 (1.83, 10.99)*
Corresponding author’s recency of epidemiology training at time of publication (≥/<5 years)	0.80 (0.27, 2.34)	2.31 (0.45, 11.86)
Corresponding author’s biostatistics training (y/n)	NA	2.38 (0.86, 6.53)
Corresponding author’s recency of biostatistics training at time of publication (≥/<5 years)	1.37 (0.45, 4.13)	2.56 (0.52, 12.51)

^a^Interdisciplinary training = a clinical degree and personal epidemiology training and personal biostatistics training

^b^Interdisciplinary teamwork = among all co-authors (including the corresponding author), at least one person has clinical training, at least one person has epidemiology training, and at least one person has biostatistics training

*Statistically significant (p < 0.05)

### Interdisciplinary teamwork

**Fig 3:**
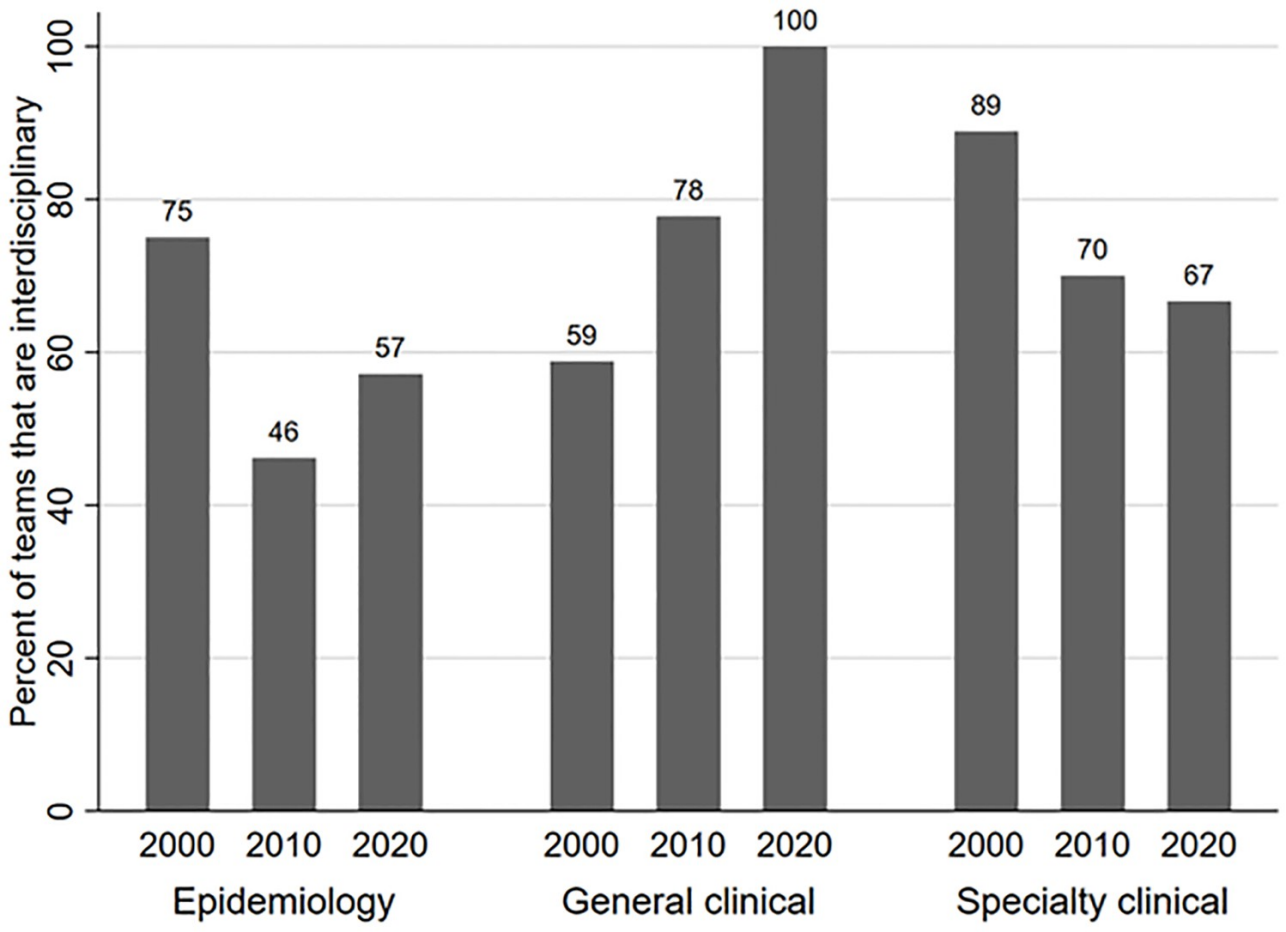
Percent of team that were interdisciplinary by type of journal and publication year. Interdisciplinary teams collectively had at least one author formally trained in each epidemiology, biostatistics, and clinical practice.

Participants frequently reported working within interdisciplinary research teams (S2 Table in S1 Text). Over 70% of our participants reported that at least one co-author (other than the respondent) had formal training in each of epidemiology (n = 74), biostatistics (n = 69), and clinical medicine (n = 76). Approximately 70% (n = 72) of participants reported that they had teams that collectively had formal training in each of the three areas (Fig 3; including the respondents’ training). Interdisciplinary teamwork (i.e., interdisciplinary team composition) was not significantly associated with year of publication, type of journal, gender, or recency of training in either epidemiology or biostatistics (p > 0.05 for each bivariate comparison, Table 2).

Interdisciplinary teams typically involved co-authors at multiple stages of the research process. Of participants who reported the research stages to which co-authors contributed, over half indicated that co-authors with epidemiological (n = 57), biostatistical (n = 42), and clinical training (n = 40) were involved in all four research stages (design, analysis, interpretation, and reporting). The year of publication, type of journal, and gender of the participant did not significantly affect whether a co-author with epidemiological or biostatistical training was involved in any of the four research stages (p > 0.05 for each bivariate comparison). However, all articles published in 2000 included someone with epidemiological training who contributed to the interpretation. Participants who reported that they had completed their epidemiological training <5 years from the date of publication all reported that they had a co-author (other than themselves) with epidemiological training who contributed to the design and interpretation of the research. The recency of participants’ biostatistical training was not significantly associated with whether a co-author trained in biostatistics contributed to any of the research stages (p > 0.05 for each comparison).

S1 and S2 Figs in S1 Text show how journal type, publication year, and gender of the participant relate to participants’ confidence in their own and their co-authors’ ability to apply epidemiological and biostatistical concepts related to their article. In general, there were no significant bivariate associations among these variables; however, participants who published in 2020 were significantly more likely to say that they were extremely confident in their epidemiological abilities whereas participants who published in 2000 or 2010 were significantly more likely to say that they were very confident (p = 0.012 and p = 0.030, respectively).

### Open-response themes

All responses to the open-ended question (n = 38) are given in the (S3 Table in S1 Text). Responses fell into three major themes. The first theme was more detailed explanation of the respondents’ training or of their co-authors’ training. Some responses in this theme mentioned specific experts and academic departments with whom the participants trained (often in lieu of formal training), some mentioned specific analytic techniques, and some addressed the sectors in which their co-authors had training. The second theme addressed the challenges in defining and reporting on academic training. Respondents mentioned that the term “biostatistics” may not be inclusive of some statistical mathematical expertise, especially for individuals who trained outside of the U.S. or in earlier time periods. Similarly, some participants commented that training in the social sciences may be an adequate replacement for training in epidemiology, depending on the specific focus of the analysis. The third theme involved reflective responses. Some participants reflected on the importance of interdisciplinary training within teams. Others reflected on how overconfidence in ones’ abilities can be problematic to the rigor of science. For example, one participant reflected self-critically on their over-confidence at the time the paper was published and mentioned that they have learned a lot since then. Finally, some responses within the third theme reflected on the administration of training programs. For example, some respondents considered the value of interdisciplinary coursework in medical schools and one respondent suggested a course that would emphasize skepticism in interpretation of research results.

## Discussion

We provided the first quantitative assessment of the interdisciplinary training and teamwork among authors publishing in top-tier epidemiological and clinical journals. Interdisciplinary training and teamwork were both common; for example, nearly three-quarters of participants with clinical training also had formal epidemiological or biostatistical training, and approximately 70% of the participants worked in teams that collectively had formal training in each of clinical medicine, epidemiology, and biostatistics. Interdisciplinary training was significantly more common among authors publishing in general clinical journals and specialty clinical journals compared to epidemiological journals. However, we did not observe significant differences in interdisciplinary training or teamwork by year of publication (2000, 2010, or 2020), gender of the corresponding author, or time between publication and the authors’ completion of formal training. Additionally, whereas neither gender nor journal type was not significantly associated with authors’ confidence in their own or their co-authors’ understanding of relevant epidemiological and biostatistical concepts, participants who published in 2020 were significantly more likely to say that they were extremely confident in their epidemiological abilities–perhaps reflecting the increasing emphasis of research methods training for clinicians [5–13]. Open-ended responses from the participants reflected challenges in defining disciplinary expertise, the value in interdisciplinary scholarship, the value of formal interdisciplinary training, and the role of (over-)confidence in estimating abilities. Overall, our findings suggest that a continued, and perhaps increased, emphasis on formal epidemiological and biostatistical training is warranted for students in academic medicine.

Just as the value of gender and racial/ethnic diversity on research teams’ impact and productivity is appreciated in the literature [23–26], the value of interdisciplinary expertise and broad co-author networks has been shown to increase the productivity of researchers and the impact of biomedical research articles [27–29]. Similarly, we found that the participants’ answers to the open-response question reflected a strong belief in the value of interdisciplinary training and teamwork. We also observed more diversity in the types of journals that individuals with interdisciplinary training published in between 2000 and 2020, a trend also reflected in our observation that journals tended to have corresponding authors with formal training in fields corresponding to the journal type. Nevertheless, and despite the recognized clinical value of understanding epidemiology and biostatistics [1, 3], a minority of participants (4/11) with clinical training but without epidemiology or biostatistics training reported that they believed they would have benefited from formal training in at least one of these areas. These results suggest that clinical educators should stress the value of gaining epidemiological and biostatistical training, along with providing the actual training.

### Strengths and limitations

Strengths of our study included the specificity of our survey instrument with regards to definitions like ‘formal training,’ our ability to look at journals from three decades in three related research areas, and our inclusion of both closed and open-ended questions. Another major strength of our study compared to most literature on authorship trends is that we obtained survey data from the corresponding authors such that we could accurately assess gender, training experience, team disciplinary composition, and other factors–rather than making assumptions based on algorithms that classify individuals. This feature of our study had three corresponding limitations–it limited our sample size, may have introduced selection bias, and may have introduced recall bias. Our small sample size meant that we focused primarily on bivariate relationships among the variables of interest. Selection bias may limit the generalizability of our findings to our source population–especially if people who were interested in our study were also more likely to have engaged in interdisciplinary training and scholarship. This selection bias may be possible since we had a low response rate (8.3%) that varied by journal type (p = 0.063) and year (p = 0.024; Fig 1). Relatedly, individuals were eligible and invited to participate more than once if they were the corresponding author of more than one target article. While this would not affect the interpretation of the results for the team composition and training, it could bias the results characterizing the corresponding authors. Based on birth year, gender, and degree type/year, this occurred 0–1 times in our sample. Recall bias could have resulted for our assessment of trends over time if individuals who published more recently more accurately reported their (and their co-author’s) training and if they were also more likely to have had formal training in the areas of relevance to this study. Other limitations include the possibility that our subjective choice of ‘top-tier’ journals did not accurately reflect the training of teams publishing in all top-tier epidemiological and clinical journals; under sampling of issues from 2020 due to COVID-19 challenges (and related possibility for misrepresentation of 2020 trends); the narrowness of our definition of interdisciplinary to teams including people trained in epidemiology, biostatistics, and clinical medicine rather than including other potentially relevant disciplines; and the lack of specificity about the terms ‘epidemiology’ and ‘biostatistics.’ Although we intended for terms ‘epidemiology’ and ‘biostatistics’ to be generally applicable to the methods, approaches, and training represented by the fields broadly, the terms (especially biostatistics) were not perceived as such by all participants. Ambiguity in these terms could lead to misrepresentation (likely underrepresentation) of the true interdisciplinarity of training and teamwork, and this bias is likely to be greater for participants who published in 2000 and 2010 compared to 2020.

Additional studies that are larger, and perhaps took advantage of web scrapping tools to identify corresponding author contact information, could address many of the limitations of our study, as well as examine academic training quality, the role of academic training on research quality, and the role of race/ethnicity and intersectional factors. Such future studies might also consider using measures of author productivity (e.g., number of publications or H-index at the time of paper publication) as a proxy for professional experience beyond self-reported education and training. Similarly, future studies could examine the role of interdisciplinary training and teamwork on article quality since proxies like journal impact factor (one of our journal inclusion criterion) are limited in their ability indicate quality, value, or prestige [30]. In the meantime, our research suggests that academic centers training clinicians should encourage interdisciplinary training–with an emphasis on gaining basic epidemiological and biostatistical skills–given the value this training seems to provide to clinician-scholars. Similarly, academic training for public health professionals should include sufficient training in basic biology and medicine concepts such that epidemiologists and biostatisticians can work effectively with clinicians.

## Conclusions

We presented the first quantitative evidence using author-reported data that articles published in top-tier medical and epidemiological journals typically are authored by scholars with interdisciplinary training and by scholars who work with interdisciplinary teams. These trends were observed across 20 years, regardless of the corresponding author’s gender or recency of training. Interdisciplinary training–such as epidemiology and biostatistics training for clinician scholars–is valued among top-tier researchers in multiple disciplines.

## Supporting information

S1 Text (DOCX)

S1 Data (CSV)
