# Supplementary material for: Academic training of authors publishing in high-impact epidemiology and clinical journals
Source: PLoS One. 2022 Jul 29;17(7):e0271159. doi: 10.1371/journal.pone.0271159 (PMC9337644; doi:10.1371/journal.pone.0271159)
Supplement: S1 Text — (DOCX) [file pone.0271159.s001.docx]

**Supporting Information**

**S1 File. Initial recruitment email.**

Dear Dr. [potential participant’s last name],

My name is REDACTED NAME FOR PEER-REVIEW and I am an REDACTED JOB TITLE at REDACTED UNIVERSITY. I am writing to invite you to participate in a research study on the academic training of researchers publishing peer-reviewed epidemiological papers. The information we collect from this survey will be used in a publication and will inform the development of resources to improve training in epidemiology and biostatistics for researchers. You are eligible to participate in this study because you are an author on [insert published paper name] published in [year] in [journal]. If you choose to participate, you will be asked to complete a survey [link] that should take no more than 5-10 minutes of your time.

If you are interested in the study, please click here [link]. If you have any questions, please feel free to reach out to me by email (REDACTED EMAIL) or phone (REDACTED PHONE NUMBER).

Additionally, the REDACTED UNIVERSITY NAME Data Protection Notice is below for you to read [participants were given the appropriate form].

Thank you very much for your help!

Sincerely,

REDACTED NAME

[Title and contact information given]

**S2 File. Follow-up recruitment email.**

Dear Dr. [potential participant’s last name],

I emailed you last week to invite you to participate in a research study on the academic training of researchers publishing peer-reviewed epidemiological papers. If you have already completed the short (5-10 minute) survey, thank you and please ignore this email. If not, we hope that you will consider participating.

You are being contacted because you are an author on [insert published paper name] published in [year] in [journal]. If you choose to participate, you will be asked to complete a short survey [link].

If you are interested in the study, please click here [*link*]. If you have any questions, please feel free to reach out to me by email (REDACTED EMAIL) or phone (REDACTED PHONE NUMBER).

Additionally, the REDACTED UNIVERSITY NAME Data Protection Notice is below for you to read [participants were given the appropriate form].

Thank you!

Sincerely,

REDACTED NAME

[Title and contact information given]

**S3 File. Survey question text.**

1. What is your gender? [participants could select one option]

- Female
- Male
- Non-Binary
- Other (Please describe)
- Prefer not to answer

2. Do you identify as transgender? [participants could select one option]

- Yes
- No
- Prefer not to answer

3. What is your year of birth (yyyy)? [participants could type a four-digit answer]
4. As of the time your paper was published, what is the most advanced degree you had been awarded above a bachelor’s degree? [participants could select one option]

- Master's
- PhD
- MD
- PhD and MD
- Other doctoral level degree
- Other
- None of the above

5. As of the time your paper was published, in what year had your most recent degree been awarded? [participants could type a four-digit answer]
6. As of the time your paper was published, in what field(s) had you obtained advanced degree(s)? (you can choose more than one)

- Epidemiology
- Biostatistics
- Medicine/clinical training
- Other public health field (please specify) [participants could type a response]
- Other [participants could type a response]

7. As of the time your paper was published, in what country (or countries) had you received your educational training? [participants could type a response]
8. As of the time your paper was published, had you ever received formal training in epidemiology? (Select all that apply)

- Yes, at least a full semester of an undergraduate-level course
- Yes, at least a full semester of a graduate-level course
- Yes, in a workshop
- Yes, in an online class
- Yes, in another place
- No
- Unsure
- Prefer not to answer

9. If answered no, unsure, or prefer not to answer to question 8: As of the time your paper was published, would you have benefited from formal training (at least a full semester course) in epidemiology? [participants could select one option]

- Yes
- No
- Unsure
- Prefer not to answer

10. If answered yes to question 8: As of the time your paper was published, in what year had you most recently obtained formal epidemiology training? [participants could type a four-digit answer]
11. As of the time your paper was published, had you ever received formal training in biostatistics? (Select all that apply)

- Yes, at least a full semester of an undergraduate-level course
- Yes, at least a full semester of a graduate-level course
- Yes, in a workshop
- Yes, in an online class
- Yes, in another place
- No
- Unsure
- Prefer not to answer

12. If answered no, unsure, or prefer not to answer to question 11: As of the time your paper was published, would you have benefited from formal training (at least a full semester course) in biostatistics? [participants could select one option]

- Yes
- No
- Unsure
- Prefer not to answer

13. If answered yes to question 11: As of the time your paper was published, in what year had you most recently obtained formal biostatistics training? [participants could type a four-digit answer]
14. As of the time your paper was published, how confident were you in your personal ability to appropriately:
    a. Apply epidemiological concepts relevant to your paper [participants could select not at all, not very, somewhat, very, or extremely]
    b. Apply biostatistical concepts relevant to your paper [participants could select not at all, not very, somewhat, very, or extremely]

In this section, we will be asking about your co-authors on the paper. Exclude your own training when answering these questions. For this section, formal training is defined as at least two full semester courses in the subject.

15. As of the time your paper was published, were any of your co-authors formally trained in epidemiology? [participants could select one option]

- Yes
- No
- Unsure

16. If answered yes to question 15: In what stage of the project did the epidemiologist(s) participate? (Select all that apply)

- Design
- Analysis
- Interpretation
- Reporting
- Other [participants could type a response]

17. As of the time when your paper was published, were any of your coauthors formally trained in biostatistics? [participants could select one option]

- Yes
- No
- Unsure

18. If answered yes to question 17: In what stage of the project did the biostatistician(s) participate? (Select all that apply)

- Design
- Analysis
- Interpretation
- Reporting
- Other [participants could type a response]

19. As of the time your paper was published, did any of your co-authors have a clinical degree? [participants could select one option]

- Yes
- No
- Unsure

20. If answered yes to question 19: In what stage of the project did the clinician(s) participate? (Select all that apply)

- Design
- Analysis
- Interpretation
- Reporting
- Other

21. If answered yes to question 19: As of the time your paper was published, did any of your clinician coauthors have formal training in epidemiology and/or biostatistics? [participants could select one option]

- Yes
- No
- Unsure

22. Prior to submitting your paper for publication in the journal it was accepted in, did anyone formally trained in epidemiology and/or biostatistics review your paper? [participants could select one option]

- Yes
- No
- Unsure

23. As of the time your paper was published, how confident were you in your co-authors’ ability to appropriately:
a. Apply epidemiological concepts relevant to your paper [participants could select not at all, not very, somewhat, very, or extremely]
b. Apply biostatistical concepts relevant to your paper [participants could select not at all, not very, somewhat, very, or extremely]
24. As a reminder, we are trying to learn about the training of authors publishing epidemiologic research. Do you have any final thoughts you would like to share with us on this topic? [participants could type a response]

**S1 Table. Bivariate associations with age of corresponding author at time of publication and having a female corresponding author.**

|  | **Age of corresponding author at time of publication** | **Having a female corresponding author** |
| --- | --- | --- |
|  | β (95% confidence interval) | odds ratio (95% confidence interval) |
| General clinical versus epidemiology journal (referent) | 1.30 (-3.13, 5.72) | 0.41 (0.16, 1.03) |
| Specialty clinical versus epidemiology journal (referent) | 2.02 (-3.14, 7.18) | 0.48 (0.17, 1.35) |
| Epidemiology journal versus general or specialty clinical versus (referent) | -1.56 (-5.58, 2.46) | 2.30 (1.00, 5.26) |
| Article year – 2000 versus 2020 (referent) | 1.19 (-4.25, 6.64) | 0.24 (0.08, 0.75) |
| Article year – 2010 versus 2020 (referent) | 2.65 (-2.77, 8.08) | 0.32 (0.10, 0.99) |
| Article year – 2010 versus 2000 (referent) | 1.46 (-2.81, 5.72) | 1.33 (0.55, 3.24) |
| Gender of corresponding author (female = reference) | 2.12 (-1.77, 6.02) | NA |
| Corresponding author’s epidemiology training (y/n) | -4.09 (-8.10, -0.08) | 1.64 (0.71, 3.79) |
| Corresponding author’s recency of epidemiology training at time of publication (<5/≥5 years) | -10.89 (-15.01, -6.76) | 1.54 (0.53, 4.49) |
| Corresponding author’s biostatistics training (y/n) | -3.90 (-8.71, 0.91) | 1.03 (0.38, 2.75) |
| Corresponding author’s recency of biostatistics training at time of publication (<5/≥5 years) | -11.14 (-15.63, -6.65) | 1.38 (0.47, 4.09) |
| Training in the U.S. (y/n) | -0.08 (-4.28, 4.12) | 1.52 (0.64, 3.61) |

**S2 Table. Training and roles of co-authors.**

|  | **Overall** | **Gender** | | **Publication year** | | | **Journal type** | | | **Time since epidemiology training** | | **Time since biostatistics training** | |
| --- | --- | --- | --- | --- | --- | --- | --- | --- | --- | --- | --- | --- | --- |
|  |  | female | male | 2000 | 2010 | 2020 | epidemiology | general clinical | specialty clinical | <5 years | ≥5 years | <5 years | ≥5 years |
|  | (n=103) | (n=46) | (n=56) | (n=42) | (n=41) | (n=20) | (n=36) | (n=42) | (n=25) | (n=20) | (n=44) | (n=17) | (n=59) |
| Co-author(s) with clinical degree, n (%) |  |  |  |  |  |  |  |  |  |  |  |  |  |
| Yes | 76 (78) | 38 (84) | 38 (73) | 26 (63) | 31 (84) | 19 (95) | 28 (80) | 28 (72) | 20 (83) | 16 (80) | 35 (81) | 13 (76) | 46 (78) |
| No | 17 (17) | 5 (11) | 12 (23) | 12 (29) | 4 (11) | 1 (5) | 5 (14) | 8 (21) | 4 (17) | 4 (20) | 4 (9) | 4 (24) | 9 (15) |
| Unsure | 5 (5) | 2 (4) | 2 (4) | 3 (7) | 2 (5) | 0 (0) | 2 (6) | 3 (8) | 0 (0) | 0 (0) | 4 (9) | 0 (0) | 4 (7) |
| Co-author(s) trained in epidemiology, n (%) |  |  |  |  |  |  |  |  |  |  |  |  |  |
| Yes | 74 (76) | 33 (73) | 41 (80) | 29 (73) | 27 (73) | 18 (90) | 29 (85) | 28 (72) | 17 (71) | 18 (90) | 36 (84) | 14 (82) | 46 (78) |
| No | 12 (12) | 7 (16) | 5 (10) | 8 (20) | 4 (11) | 0 (0) | 2 (6) | 5 (13) | 5 (21) | 2 (10) | 3 (7) | 2 (12) | 6 (10) |
| Unsure | 11 (11) | 5 (11) | 5 (10) | 3 (8) | 6 (16) | 2 (10) | 3 (9) | 6 (15) | 2 (8) | 0 (0) | 4 (9) | 1 (6) | 7 (12) |
| Co-author(s) trained in biostatistics, n (%) |  |  |  |  |  |  |  |  |  |  |  |  |  |
| Yes | 69 (70) | 32 (71) | 36 (69) | 27 (66) | 26 (70) | 16 (80) | 22 (63) | 28 (72) | 19 (79) | 17 (85) | 30 (70) | 12 (71) | 42 (71) |
| No | 15 (15) | 7 (16) | 8 (15) | 8 (20) | 5 (14) | 2 (10) | 7 (20) | 5 (13) | 3 (13) | 3 (15) | 6 (14) | 5 (29) | 6 (10) |
| Unsure | 14 (14) | 6 (13) | 8 (15) | 6 (15) | 6 (16) | 2 (10) | 6 (17) | 6 (15) | 2 (8) | 0 (0) | 7 (16) | 0 (0) | 11 (19) |
| Formally trained epidemiologist and/or biostatistician reviewed paper, n (%) |  |  |  |  |  |  |  |  |  |  |  |  |  |
| Yes | 58 (59) | 29 (64) | 28 (54) | 19 (48) | 22 (58) | 17 (85) | 19 (54) | 24 (62) | 15 (63) | 14 (70) | 27 (63) | 11 (65) | 36 (61) |
| No | 32 (33) | 13 (29) | 19 (37) | 17 (43) | 13 (34) | 2 (10) | 12 (34) | 12 (31) | 8 (33) | 6 (30) | 10 (23) | 6 (35) | 17 (29) |
| Unsure | 8 (8) | 3 (7) | 5 (10) | 4 (10) | 3 (8) | 1 (5) | 4 (11) | 3 (8) | 1 (4) | 0 (0) | 6 (14) | 0 (0) | 6 (10) |
| Confidence in co-authors’ ability to apply epidemiology concepts to paper, n (%) |  |  |  |  |  |  |  |  |  |  |  |  |  |
| Not at all/Not very | 8 (8) | 1 (2) | 7 (14) | 5 (13) | 3 (8) | 0 (0) | 1 (3) | 6 (15) | 1 (4) | 0 (0) | 1 (2) | 2 (12) | 4 (7) |
| Somewhat | 12 (13) | 7 (16) | 5 (10) | 6 (15) | 6 (16) | 0 (0) | 5 (15) | 5 (13) | 2 (9) | 0 (0) | 3 (7) | 0 (0) | 5 (8) |
| Very | 30 (31) | 17 (39) | 13 (25) | 10 (25) | 13 (35) | 7 (37) | 12 (35) | 9 (23) | 9 (39) | 7 (35) | 17 (40) | 7 (41) | 20 (34) |
| Extremely | 46 (48) | 19 (43) | 26 (51) | 19 (48) | 15 (41) | 12 (63) | 16 (47) | 19 (49) | 11 (48) | 13 (65) | 22 (51) | 8 (47) | 30  (51) |
| Confidence in co-authors’ ability to apply biostatistics concepts to paper, n (%) |  |  |  |  |  |  |  |  |  |  |  |  |  |
| Not at all/Not very | 5 (5) | 1 (2) | 4 (8) | 3 (8) | 2 (5) | 0 (0) | 3 (9) | 2 (5) | 0 (0) | 0 (0) | 2 (5) | 0 (0) | 3 (5) |
| Somewhat | 18 (19) | 8 (18) | 10 (20) | 8 (20) | 8 (22) | 2 (11) | 5 (15) | 9 (23) | 4 (17) | 2 (10) | 6 (14) | 3 (18) | 10 (17) |
| Very | 33 (34) | 20 (45) | 13 (25) | 13 (33) | 12 (32) | 8 (42) | 14 (41) | 10 (26) | 9 (39) | 8 (40) | 16 (37) | 8 (47) | 20 (34) |
| Extremely | 40 (42) | 15 (34) | 24 (47) | 16 (40) | 15 (41) | 9 (47) | 12 (35) | 18 (46) | 10 (43) | 10 (50) | 19 (44) | 6 (35) | 26 (44) |

**S3 Table.** **Open-ended responses about training.** Participants responded to the prompt: “As a reminder, we are trying to learn about the training of authors publishing epidemiologic research. Do you have any final thoughts you would like to share with us on this topic?”

| **Theme** | **Response** |
| --- | --- |
| Respondent's training | this paper was published while I was in process of defending my PhD dissertation in Epidemiology, and my doctorate degree was awarded less than 2 months after this paper came out. |
|  | Work conducted as a PhD student, supervised by world leader in longitudinal data analysis [redacted name and degree] |
|  | My training is not in biostats/epi but in another field where the math rigor, stats, and modeling is at a more rigorous and technical level than what is formally taught in public health or medical schools |
|  | After about 5 years from my PhD I worked for a year with [redacted name]. I think that was probably the best training I could have had! I also subsequently worked with [redacted name], so again learnt a lot |
|  | I basically trained myself in clinical epidemiology and biostatistics. |
|  | I think the emphasis on formal training in this survey is problematic. I have systematically avoided courses in biostatistics and epidemiology and instead have learned through close collaborations with researchers who have such training. For me that is a more effective way of learning. I think I'm not the only one. |
|  | My understanding of epidemiology and biostatistics is largely from working in a team of doctorate level epidemiologists and biostatisticians over approximately 30 years. |
|  | Since that paper was published over 20 years ago, I have received much more research training but still lag in epidemiology skills. |
|  | We all were quite well trained in clinical epidemiology in the beginning in the [redacted year and university] and I have always found it extremely helpful to have a sound epidemiological background although having a clinical background. thereafter I personally had some more intense course on Regression analysis which has also been extremely helpful |
|  | This was a fairly straightforward pooled analysis. In retrospect, I would have used a random effects meta-analysis statistical approach. But either way, the conclusion would have been the same because of the extremely low incidence of the outcome of interest. |
| Co-authors' training | We formed a multi-author multidisciplinary team that specifically included a highly qualified epidemiologist and individuals with extensive experience in survey design, as well as public health and clinical experience. I don't think it is possible for any one individual to hold all the expertise needed for this sort of project. |
|  | This paper happened to have extensive Epi training and mix of related training: [redacted list of training of individual authors] |
|  | the paper was part of a PhD training and became a chapter in a PhD thesis. All co-authors were members of the department of Epidemiology and Biostatistics |
|  | My doctorate is in epidemiology, one of the co-authors is a doctoral candidate and we're both trained in biostats as part of the epi PhD. I would guess that you're trying to understand whether papers with epi and analytical content consult with trained epidemiologists and biostatisticians. Given that, when the affiliation of two of the authors is a department of epidemiology in a school of public health then there is an increased likelihood that those authors will have training in epi |
|  | This paper didn't have a co-author who was primarily an epidemiologist or a biostatistician, but the senior author was a clinician who had MPH training in epi and biostats, as I did. |
|  | You have picked an interesting example where, unusually for our group, there was no formally trained statistician on the team. The three clinicians [redacted specialties] were all very experienced researchers and two of them had done additional short courses in epidemiology and in statistics but, as far as I can recall, did not have postgraduate qualifications in either. I think this style of collaboration at an individual level is now much rarer - projects like this would now expect to have formal statisticians and information scientist on board. |
|  | my coauthors had training in health services research and pharmaceutical health outcomes research. I think training in these fields is also very applicable for applied epidemiology/policy analysis and you may be under-representing this expertise if just focusing on formal epidemiology and biostatistical training. |
|  | That paper represented an active collaboration between scientists working in a government agency and academic colleagues from academia. It was teamwork. Authors in public health need to learn how to work in multi-disciplinary teams. |
|  | I am fortunate to be part of a very diverse and highly experienced team |
| Challenges in reporting on training | Epidemiology overlaps a *lot* with quite a few other social sciences!!!!!!! |
|  | You talk about Biostatistics but I studied Statistics. Many older statisticians like me studied at a time when the term hadn't been invented. Indeed, the term is particularly American - I am now Prof of [redacted field] in [redacted non-US country] and would never call myself a biostatistician. Your survey might, thus, misrepresent the prevalence of (bio)statisticians |
|  | You need to *DEFINE* biostatistics, and possible even EPIDEMIOLOGY before starting to ask question about the topics. For example, a Master's degree in mathematics focusing on mathematical statistics, is that biostatistics or not!? Additionally, it's unclear why you probe into a paper published 10 years ago, it's very hard to remember exactly what happened in what order so long ago. |
|  | my coauthors had training in health services research and pharmaceutical health outcomes research. I think training in these fields is also very applicable for applied epidemiology/policy analysis and you may be under-representing this expertise if just focusing on formal epidemiology and biostatistical training. |
| Reflections on interdisciplinarity | That paper represented an active collaboration between scientists working in a government agency and academic colleagues from academia. It was teamwork. Authors in public health need to learn how to work in multi-disciplinary teams. |
|  | We formed a multi-author multidisciplinary team that specifically included a highly qualified epidemiologist and individuals with extensive experience in survey design, as well as public health and clinical experience. I don't think it is possible for any one individual to hold all the expertise needed for this sort of project. |
|  | Training is essential. Too many people assume they know how to interpret data and as a result, the quality of many papers suffer. It’s as complex as specialty training in [redacted clinical field]! |
|  | I am fortunate to be part of a very diverse and highly experienced team |
| Reflections on the role of confidence | At the time, I was very young and overconfident. As it was twenty years ago and I have learned a lot since then, I would not be surprised to find errors in the paper, if I were to read it again today. |
|  | these questions are a bit subjective. e.g., someone with lots of confidence will check 'extremely' whereas others who are less confident will not. |
| Reflections on training programs | Most US medical school provide several curriculum lectures in Epidemiology. I[t] provides recognition, but not expertise. |
|  | The problem with statistics teaching in medical schools that I know is that it tends to be too theoretical and too early (you forget!) to be of real use when initiating research. |
|  | All students at schools of public health should be required to take a course or seminar on the importance of skepticism in the interpretation of one's research |
|  | I have found having formal epidemiology and biostatistics training to be extremely useful in publishing the research that I conduct. |
|  | I am not sure how someone who does not have epidemiology training could publish good research. This point seems obvious. Why do you need a survey to figure this out |
|  | We all were quite well trained in clinical epidemiology in the beginning in [redacted year and university] and I have always found it extremely helpful to have a sound epidemiological background although having a clinical background. thereafter I personally had some more intense course on Regression analysis which has also been extremely helpful |
|  | This was a fairly straightforward pooled analysis. In retrospect, I would have used a random effects meta-analysis statistical approach. But either way, the conclusion would have been the same because of the extremely low incidence of the outcome of interest. |
|  | I think the emphasis on formal training in this survey is problematic. I have systematically avoided courses in biostatistics and epidemiology and instead have learned through close collaborations with researchers who have such training. For me that is a more effective way of learning. I think I'm not the only one. |
| Other | The paper you are requesting information on was published in 2000, developed prior to that. It was a small sample trial that was uncontrolled. Not worth a lot of attention in today's world, from a statistical or design standpoint. |
|  | The study that you are referring to was not epidemiological in its nature. |
|  | this was a narrative review, not a scientific study. |
|  | You selected one of my papers that was published in [redacted name of epidemiology journal]. I hope your research is capturing authors of epi papers outside of the mainstream epi journals. |
|  | I would be more confident that I had answered the questions correctly if you had picked a more recent paper. |
|  | Some coauthors were put on there because of their connection to the funding source, although they did contribute. |

**S1 Fig. Confidence in personal epidemiological and biostatistical abilities. Panels A and B show the percent of participants with different levels of confidence in their personal epidemiological (A) and biostatistical (B) abilities by journal type and publication year. Panels C and D show the percent of participants with different levels of confidence in their personal epidemiological (C) and biostatistical (D) abilities by journal type, publication year, and gender.**

**S2 Fig. Confidence in co-author epidemiological and biostatistical abilities. Panels A and B show the percent of participants with different levels of confidence in their co-authors’ epidemiological (A) and biostatistical (B) abilities by journal type and publication year. Panels C and D show the percent of participants with different levels of confidence in their co-authors’ epidemiological (C) and biostatistical (D) abilities by journal type, publication year, and participant gender.**
